# Characterization of bovine immortalized luteal endothelial cells: action of cytokines on production and content of arachidonic acid metabolites

**DOI:** 10.1186/1477-7827-9-27

**Published:** 2011-02-24

**Authors:** Anna J Korzekwa, Gabriel Bodek, Joanna Bukowska, Agnieszka Blitek, Dariusz J Skarzynski

**Affiliations:** 1 Department of Reproductive Immunology and Pathology, Institute of Animal Reproduction and Food Research, Polish Academy of Sciences, 10-747 Olsztyn, Poland; 2 In vitro and Biotechnology Laboratory, Institute of Animal Reproduction and Food Research, Polish Academy of Sciences, 10-747 Olsztyn, Poland; 3 Department of Hormonal Action Mechanisms, Institute of Animal Reproduction and Food Research, Polish Academy of Sciences, 10-747 Olsztyn, Poland

## Abstract

**Background:**

The interactions between luteal, vascular endothelial, immune cells and its products: steroids, peptide hormones, prostaglandins (PGs), growth factors and cytokines play a pivotal role in the regulation of corpus luteum (CL) function. Luteal endothelial cells undergo many dynamic morphological changes and their action is regulated by cytokines. The aims are: (1) to establish *in vitro *model for bovine luteal endothelial cells examination; (2) to study the effect of cytokines: tumor necrosis factor alpha (TNFalpha) and interferon gamma (IFNgamma) on cell viability, leukotrienes (LTs) and PG synthases, and endothelin-1 (EDN-1) mRNA, protein expression and their secretion in bovine immortalized luteal endothelial (EnCL-1) cells.

**Methods:**

The primary cultures of bovine luteal endothelial cells were immortalized by transfection with vector carrying the Simian virus 40 T-antigen (SV40 T-ag) sequence. Expression of SV40 T-ag gene in EnCL-1 cells was confirmed by RT-PCR and immunofluorescence staining showed the presence of endothelial cell markers: VE-cadherin and von Willebrand factor. EnCL-1 cells were stimulated by TNFalpha with IFNgamma (50 ng/ml each) for 24 h. Cell viability, mRNA expression (*real time RT-PCR*), protein expression (*western blotting*) for LTC4 synthase (LTC4S), LTA4 hydrolase (LTA4H), PGE2 and PGF2alpha synthases and endothelin-1 (EDN-1), and levels of LTs (B4 and C4) and PGs (E2 and F2alpha) and EDN-1 in the medium (*EIA*) were evaluated.

**Results:**

We received immortalized luteal endothelial cell line (EnCL-1). Cytokines did not change EnCL-1 cell viability but increased mRNA expression of LTC4S, LTA4H, PGE2 and PGF2alpha synthases and EDN-1. EDN-1/2/3, LTC4 and PGF2alpha synthases protein expression were elevated in the presence of TNFalpha/IFNgamma, and accompanied by increased EDN-1, LTC4 and PGF2alpha secretion. Cytokines had no effect on PGES and LTA4H protein expression, and PGE2 and LTB4 release.

**Conclusions:**

TNFalpha and IFNgamma modulate EnCL-1 cell function. Moreover, established EnCL-1 cell line appears to be a good model for investigating the molecular mechanisms related to cytokines action and aa metabolites production in cattle.

## Background

Although corpus luteum (CL) is a transient gland, it is one of the most vascularized tissues in the body [1], with endothelial cells representing greater than fifty percent of the total cells [2,3]. Angiogenesis is critical to CL development, whereas endothelial cells decline occurs during luteolysis [4]. On the other side, endothelial cells play a crucial role in a complex processes of tumor neovascularization [5], including CL cancers [6]. Because of these important and multiplex functions of endothelial cells in CL vascularity, the establishment of an experimental model of immortalized endothelial cells from bovine CL is a prerequisite for the study of cellular and molecular mechanism in this tissue. So far, the majority of studies have been conducted on fresh isolated or refrozen aliquots of bovine primary luteal endothelial cells [4,7-9] or cell line received not directly from CL [10]. Immortalized endothelial cells have been characterized in several kinds of bovine tissues, such as the pulmonary and coronary arteries, nevertheless no bovine luteal endothelial cell line is available [10]. There is a possibility that surface antigens and/or genetic programming differs for endothelial cells derived from various tissues, beside each kind of cell is strictly species dependent [11]. Thus, the stable bovine luteal endothelial cell line with determined fenotype and genotype would be the convenient and useful model for the future study.

Among mediators of interactions between different types of CL cells, including endothelial cells, the universal factors are immune cells and their secreted products, cytokines [12-14]. Endothelial cells are capable of tumor necrosis factor α (TNFα) synthesis and secretion [15]. Depending on the immediate microenvironment, TNFα may stimulate cell proliferation or induce apoptosis of luteal endothelial cells [4]. TNFα action in the bovine CL is a dose dependent: a low concentration of TNFα stimulates *in vivo *luteolytic factors, as well as induces apoptosis; whereas the high concentration of TNFα stimulates a survival pathway [16-19]. Moreover, TNFα induced apoptosis in cultured bovine luteal endothelial cells [20]. TNFα effect in the ovary was found to be more effective when TNFα acted synergistically with interferon γ (IFNγ; [13,19,21,22]). Reasonable is the generation of stable *in vitro *luteal endothelial cell culture for investigating the complex signaling pathway and transcriptional mechanisms regulated by cytokines in physiological and pathophysiological conditions in cattle.

The proper vascularization and endothelial cell activity *per se *are essential for normal CL function [23]. The effect of prostaglandins (PGs) on the vascularity of bovine CL is well known [9,21,23-26]. The ovarian blood flow has been shown to increase after PGE_2 _administration and decrease during spontaneous and PGF_2α _induced luteolysis in cows [27]. An acute increase in the luteal blood flow occurs as the first step of luteolysis in response to PGF_2α _[3]. Both the density and the number of blood vessels were higher in CLs obtained after PGF_2α _administration than in those without PGF_2α _treatment [21], which indicate that the number of blood vessels with smooth muscle in the regressing CL increased as a result of loosing steroidogenic cells and capillaries. A mitogenic effect and increased proliferation were observed after PGF_2α _treatment in bovine dispersed luteal endothelial cells [23]. Moreover, PG receptors, as well as leukotriene (LTs - other metabolites of arachidonic acid - aa) receptors are present on endothelial cells [23,26], which indicate that the endothelial cells of bovine CL are target for PGs and LTs. Leukotrienes are commonly known as the potential inflammatory factors that cause edema in respiratory tract diseases, but they also play the important role in reproduction and may enhance the action of PGs [28]. We hypothesize that cytokines are involved in aa metabolites action in bovine luteal endothelial cells.

The main goal of this study was to determine the effect of cytokines on aa metabolites action in bovine immortalized luteal endothelial cells. We examined: (i) the viability of EnCL-1 cells, (ii) mRNA expression for LTA_4 _hydrolase (LTA4H), LTC_4 _synthase (LTC4S), PGE_2 _and PGF_2α _synthases (PGES and PGFS) and endothelin-1 (EDN-1), (iii) protein expression for LTA4H, LTC4S, PGES, PGFS and EDN-1/2/3 and (iv) accumulation of LTB_4 _and C_4_, PGE_2 _and F_2α _and EDN-1 in the culture medium after TNFα/IFNγ stimulation.

## Methods

### Collection of CL for in vitro experiments

Healthy, normally cycling Holstein/Polish Black and White (75/25%; respectively) cows (3-4 lactation) were used for the collection of the ovaries with CL (in total 4 animals). The animals were eliminated by the owners (Experimental Animal Farm of Polish Academy of Sciences in Baranowo; Poland) from the dairy herds because of their lower milk production. The estrus of the cows was synchronized twice using an analogue of PGF_2α _(dinoprost, Dinolytic; Upjohn - Pharmacia N.V.S.A., Belgium) injections with an 11-day interval as recommended by a vendor. The onset of the estrus was determined by a veterinarian via *per rectum *USG examination using a DRAMINSKI ANIMALprofi Scanner [29] and confirmed by observing the signs of estrus (i.e. vaginal mucus, standing behavior). The onset of estrus was determined as Day 0. Only cows with signs of estrus were chosen for the study. Animals were slaughtered on Day 8-12 of the estrous cycle and the ovaries were obtained within 20 min of the exsanguinations and transported on ice to the laboratory.

### Luteal endothelial cells isolation

Endothelial cells isolation was proceeded according to the method described previously in details [8,26] using a Dynabeads kit (140.03 Dynabeads^® ^M-450; Invitrogen, Oregon, USA). Briefly, the Dynabeads were coated with the specific antigen-lectin (BS-1; L 2380 Sigma; Chemical Co., St Louis, MO, USA). The beads coated with endothelial cells were attracted by a magnet to the well of the tube and the supernatant was removed. After washing with PBS, 1 ml of 0.1 M fucose (T 2252 Sigma) solution was added to break the connection of endothelial cells with beads. The free beads were then attracted by a magnet to the well of the tube and the supernatant with endothelial cells was collected. The obtained cell suspension contained more than 85% of luteal endothelial cells and only a few steroidogenic CL cells. The cells were suspended in Dulbecco's Modified Eagle's medium (DMEM/Ham's F-12; 1: 1(v/v); D 2906 Sigma) in a 3 ml culture flask in a humidified incubator at 37.5°C in 5% CO_2_/95% air atmosphere. After third passage the cells were trypsinized and placed at the concentration of 2 × 10^5 ^cells/ml into a 24-well culture plate. After 24-48 h of culture cells reached confluence and were proceeded the procedure of immortalization.

#### Experimental procedures

##### Experiment 1. Establishment of immortalized bovine endothelial cell (EnCL-1) line and its phenotype characterization

The primary cultures of endothelial cells were immortalized by transfection with the vector carrying a Simian virus 40 T-antigen (SV40 T-ag) sequence. Lipofectamine LTX (11514-015, Invitrogen) was used as a transfection agent according to manufacturer's instruction. The fast proliferating colonies were selected and initially scanned for the presence of transfected vector - SV40 T-ag. Expression of SV40 T-ag gene was further confirmed by RT-PCR in selected passages of the cells (13, 18, 23, 31, 40, 50^th^). The selected immortalized cell line call EnCL-1 was cultured for next 50 passages without any sign of senescence. After 13^th ^passage every 5^th ^passage of EnCL-1 cells were pulled and frozen in -80°C in cryotube portions (1.5 ml at the concentration 2.0 × 10^5^/ml). Viability of unfrozen cells was higher than 85% as assessed by trypan blue dye exclusion. Characterization of EnCL-1 cell line was provided by fluorescence morphology and cytoplasmatic protein - von Villenbrand factor and cell to cell adhesion - VE-cadherin in selected passages of EnCL-1 cells (13, 18, 23, 31, 40, 50^th^).

##### Experiment 2. Effect of cytokines on production and secretion of Arachidonic Acid metabolites in immortalized bovine endothelial cells (EnCL-1)

The concentration of TNFα and IFNγ (each in the dose: 50 ng/ml), and exposure time (24 h) were determined on the basis of previous studies [18,19,22,26,30]. All treatments were conducted in triplicates, four experiments were performed.

##### Experiment 2.1. Effect of TNFα and ifNγ on the viability of immortalized bovine luteal endothelial cells (EnCL-1)

The aim of the experiment was to evaluate the percentage of live cells after stimulation with studied cytokines comparing with non-treated cells. The EnCL-1 cells (13^th ^passage) were adjusted to 2.0 10^5^/ml of medium: DMEM supplemented with 5% calf serum (F 7524 Sigma) and 20 μg/ml gentamycin. Cells were cultured in 96-culture plates in a humidified incubator at 37.5°C in 5% CO_2 _and 95% air atmosphere. After 24 h, the cells were washed with serum-free DMEM and the medium was replaced by fresh medium: DMEM/Ham's F-12 supplemented with 0.1% (w/v) BSA and containing 20 μg/ml gentamycin. Then, the cells were treated simultaneously with TNFα (recombinant human TNFα: HF-13; Dainippon Pharmaceutical Co., Ltd., Osaka, Japan) and IFNγ (PBP007; AbD Serotec, Biokom, Warsaw, Poland) for 24 h. Cell viability (TOX-1; Sigma) was measured using commercially available colorimetric assay kits according to the manufacturer"s instructions as previously described [18].

##### Experiment 2.2. Effect of TNFα and ifNγ on mRNA and protein expression of LTC_4 _synthase, LTA_4 _hydrolase, PGE_2 _synthase, PGF_2α _synthase and endothelin-1 in bovine endothelial immortalized cells (EnCL-1)

The aim of the experiment was to examine whether TNFα and IFNγ affect mRNA and protein expression of selected factors. Dispersed, unfrozen EnCL-1 cells (13^th ^passage) were seeded at 2.0 × 10^5 ^viable cells in 1 ml of cultured medium in 24-well culture plates (3524, Costar). After 18 h of culture in DMEM medium containing 5% CS, the medium was replaced with DMEM containing 0.1% BSA with or without TNFα/IFNγ.

mRNA expression was quantitavely measured by real-time RT-PCR for LTC4S, LTA4H, PGES, PGFS and EDN-1 and protein expression was measured by western blotting for LTC4S, LTA4H, PGES, PGFS and EDN-1/2/3 as previously described [26,30]. The sequences of used primers are described in Table 1. The cells were disrupted with TRIZOL Reagent (15596, Invitrogen) and frozen at -80°C until processed for RNA isolation and Reverse Transcription-Polymerase Chain Reaction (RT-PCR). Separately, the cells were frozen at -80°C until processed for protein isolation.

**Table 1:** Oligonucleotide sequences used for RT-PCR

Gene	Oligonucleotide sequences	Product size (bp)	GenBank/REFERENCES
**GAPDH**	F 5'-CACCCTCAAGATTGTCAGCA-3' R 5'-GGTCATAAGTCCCTCCACGA-3'	103	BC102589
**LTA4H**	F 5'-CCCTAAAGAACTGGTGGCACT-3' R 5'-GACTTTTCCACCTGCTCTTTC-3'	240	NM00103428 [46]
**LTC4S**	F 5'-CCTGCTGCAAGCCTACTTCT-3' R 5'-GTTCACTTGGGCTCGGTAGA-3'	137	NM001046098
**PGES**	F 5'-ATCGTGACGGTCCGTCTCTAA-3' R 5'-GCCCTTTGAGATTGTGACAGG-3'	158	NM174443 [47]
**PGFS**	F 5'-TGTGGTGCACGTATCACGACA-3' R 5'-AATCACGTTGCCGTCCTCATC-3'	169	S54973 [47]
**EDN-1**	F 5'-CAAATGCATCCTGCCTGGTC-3' R 5'-ATTGCCACCCCCATAGAGGA-3'	178	X52740 [25]
**SV40 T-ag**	F 5'-GCAATCGAAGCAGTAGCAATC-3' R 5'-CAGCTAATGGACCTTCTAGG-3'	385	SV40gp6 [48]

##### Experiment 2.3. Effect of TNFα and ifNγ on prostaglandins, leukotrienes and endothelin-1 release by EnCL-1 cells

The aim of the experiment was to study the effect of TNFα and IFNγ on release of PGE_2_, PGF_2α_, LTB_4_, LTC_4 _and EDN-1 by EnCL-1 cells.

After incubation, media from Experiment 2.2 were collected into tubes containing 10 μl of stabilizer [0.3 M EDTA and 1% aspirin, A 2093; Sigma] for each 500 μl of medium and stored at - 20°C until EIA determinations.

### Total RNA isolation

Total RNA was extracted from EnCL-1 cells using TRIZOL Reagent according to the manufacturer's instructions. One microgram of each sample of total RNA was reverse transcribed using the SuperScript First-Strand Synthesis System for RT-PCR (11904-018 Invitrogen), as described in the supplier's protocol.

### Conventional PCR

mRNA expression of the SV40 T-ag was confirmed by conventional PCR using primers for SV40 T-ag detailed in Table 1. The EnCL-1 cells cDNA was amplified with JumpStar™ REDTaq™ ReadyMix PCR Reaction Mix (P 0982; Sigma). The PCR conditions were as follows: 3 min, 95 °C and 30 sec, 58°C, and 30 sec 72°C for 30 cycles. The samples were electrophoresed on 1,5% agarose gel containing ethidium bromide.

### Immunofluorescence staining

EnCL-1 were plated in 2- and 4- well chamber slides at a concentration 1 × 10^5 ^cells/ml and after 24 h the cells were fixed with 4% paraformaldehyde, washed 3x with PBS and blocked with PAV/10% NDS (normal donkey serum) 1 h. Then, the cells were incubated overnight with primary antibodies specific to von Willenbrand factor and VE-cadherin. Next, the cells were washed 3x with PBS and incubated 1 h room temp. with secondary antibodies conjugated with cyanine 3 (CY^3^; Jackson ImmunoResearch, West Grove, PA). Furthermore, the cells were counterstained with DAPI UltraCruz™ Mounting Medium (sc-24941; Santa Cruz Biotechnology). EnCL-1 cells were visualized with confocal imaging using a Nicon C1 confocal microscope.

### Real-time PCR quantification

Quantitative fluorescence real-time PCR was performed using the Applied Biosystems 7300 System (850 Lincoln Centre Drive Foster City, CA 94404 USA) with a SYBR Green PCR master mix (4367659; Power SYBR^® ^Green PCR Master Mix; Applied Biosystems) following the manufacturer's instructions. Real-time PCR (25 μl) included 12.5 μl SYBR Green PCR Master Mix, 0.5 μM sense and antisense primers each, and reverse-transcribed cDNA (1 μl of cDNA). Primer sequences are detailed in Table 1. For quantification, standard curves consisting of serial dilutions of the appropriate purified cDNA were plotted. Amplification was proceeded by an initial denaturation step (15 min at 95°C). The PCR programs for each gene were performed as follows: 40 cycles of denaturation (15 sec at 95°C), annealing (30 sec at 56°C), and elongation (60 sec at 72°C). After PCR, melting curves were acquired by stepwise increases at a temperature of 50-95°C to ensure that a single product was amplified in the reaction. The data obtained from the real-time RT-PCR for LTC4S, LTA4H, PGES, PGFS and EDN-1 were normalized against GAPDH. Control reactions in the absence of reverse transcriptase were performed to test for genomic DNA contamination. The specify of the PCR products for examined genes was confirmed by gel electrophoresis and sequencing. The sequence homology obtained in the experiment was 99%. Dissociation curve analysis was performed after each real-time experiment to confirm the presence of only one product and the absence of the formation of primer dimmers. The threshold cycle number (Ct) for each tested gene X was used to quantify the relative abundance of the gene; arbitrary units were calculated as: 2^-{∆}Ct ^= 2^- (Ct target gene-Ct housekeeping gene)^.

### EIA determination

Concentration of PGE_2 _in culture media was determined by EIA as previously described [31]. The PGE_2 _standard curve ranged from 0.07 to 20 ng/ml and the ID50 of the assay was 1.25 ng/ml. The intra- and inter-assay CV averaged 6.9% and 9.7% respectively. Concentration of PGF_2α _in culture media was determined by EIA as described previously [31].

The PGF_2α _standard curve ranged from 0.07 to 20 ng/ml, and the ID50 of the assay was 1.82 ng/ml. The intra- and inter-assay CV were 7.4% and 11.6% respectively.

Concentration of EDN-1 in culture media was determined by EIA using commercially kit (385131, Endothelin-1 EIA Kit, Cayman, Cayman Chemical, Ann Arbor, MI, USA). The EDN-1 standard curve ranged from 0.39 to 250 pg/ml and the ID50 of the assay was 7.8 pg/ml. The intra- and inter-assay CV was 10.0%.

The concentrations of LTB_4 _and C_4 _in culture media were determined using commercially available EIA kits (520211, Leukotriene C_4 _EIA Kit, 520111, Leukotriene B_4 _EIA Kit; Cayman) according to Korzekwa et al. [19]. The LTB_4 _standard curve ranged from 1.96 to 1000 pg/ml, and the effective dose for 50% inhibition (ID50) of the assay was 2.5 pg/ml. The intra- and inter-assay coefficients of variation were on average 4.1% and 6.2%, respectively. The LTC_4 _standard curve ranged from 0.98 to 500 pg/ml and the effective dose for 50% inhibition (ID50) of the assay was 1.85 pg/ml. The intra- and inter-assay CV were on average 4.9% and 7.4%, respectively.

### Western blot analysis

The equal amounts (30 μg) of protein were dissolved in SDS gel-loading buffer (50 mM Tris-HCl, pH 6.8; 4% SDS, 20% glycerol and 2% β-mercaptoethanol), heated to 95°C for 4 min and separated on 10% (for GAPDH, PGFS, LTA4H, EDN-1/2/3) and 15% (for LTC4S and mPGES-1) SDS-PAGE. Separated proteins were electroblotted onto 0.2 μm nitrocellulose membrane in transfer buffer (20 mM Tris-HCl buffer, pH 8.2; 150 mM glycine, 20% methanol). After blocking in 5% non-fat dry milk in TBS-T buffer (Tris-buffered saline, containing 0.1% Tween-20) for 1.5 h at 25.6°C, the membranes were incubated overnight with 1:2000 anti-lung-type PGFS antiserum antiserum [32], or 1:1000 polyclonal anti-mPGES-1 antibodies (160140; Cayman) or 1:1000 polyclonal LTC_4 _synthase antibody (sc-20108; Santa Cruz Biotechnology, Inc, Heidelberg, Germany) or 1:1000 LTA_4 _hydrolase antibody (ab23677; Abcam, Cambridge, UK) or 1:200 EDN 1/2/3 (sc-98727; Santa Cruz Biotechnology, ET-1/2/3 = EDN-1, EDN-2, EDN-3) at 4°C. Protein expression of GAPDH (G 8795; Sigma, glyceraldehyde-3-phosphate dehydrogenase) as a reference was used. Subsequently, proteins were detected by incubating the membrane with 1:20000 dilution of secondary polyclonal anti-rabbit or anti-goat alkaline phosphatase-conjugated antibodies (A 3687; A 3562 Sigma) for 1.5 hour at 25.6°C. Immune complexes were visualized using standard alkaline phosphatase visualization procedure. Western blots were quantitated using Kodak 1D program (Eastman Kodak, Rochester, NY, USA).

### Statistical analysis

The statistical significance of differences between the control and treated groups were analyzed by one-way ANOVA followed by t-student's post-hoc test (ANOVA; GraphPAD PRISM Version 5.00, San Diego, CA; USA), if the initial ANOVA was significant (P < 0.05).

## Results

### Experiment 1. Establishment of immortalized bovine endothelial cell (EnCL-1) line and its phenotype characterization

**Figure 1:**
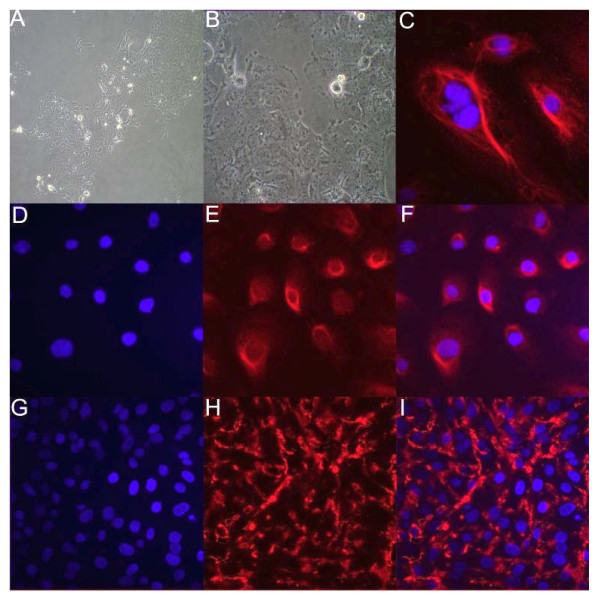
**Characterization of morphology and cytoplasmatic protein - von Villenbrand factor and cell to cell adhesion - VE-cadherin in EnCL-1 cells**. Cells reveal cobblestone morphology and tendency to form monolayer on Petri dish (A-B, mag. x10, x40). Localization of cytoplasmatic protein - vWF (panel E, mag. x10, x40) and expression of VE-cadherin (panel H, mag. x10, x40). Cells (13^th ^passage) were stained with specific antibodies and detected by secondary antibodies labeled with fluorescent dyes, and analyzed by confocal microscopy. The DNA was counterstained with DAPI (blue, panels D, G). Panels C, F and I merged pictures of two fluorescent dyes. Panel C mag. x100.

**Figure 2:**
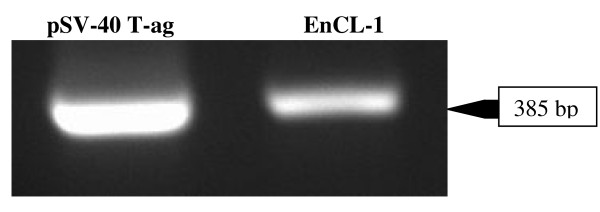
**Expression of SV40 T-ag mRNA in EnCL-1 cells by RT-PCR**. Left - SV40 Tag positive control, Right-EnCL-1 cells.

Selected immortalized endothelial cell line (EnCL-1) was cultured till 50 passages without any sign of senescence, which allowed to receive clear immortalized line of cells with homogenous morphology and genotype, although from 10 passage the line of cells has no any percentage of primary luteal endothelial cells. Phase contrast microscopy revealed that immortalized EnCL-1 cells grew as confluent monolayers with typical cobblestone morphology of primary endothelial cells. These cells (13^th ^passage) were homogenous, polygonal and had characteristic ovoid nuclei (Figure 1a,b). Moreover, immunofluorescence staining revealed the presence of endothelial cell markers: von Willebrand factor (Figure 1C,E,F) and VE-cadherin (Figure 1H,I) in EnCL-1 cells. All isolated colonies expressed transfected vector. Expression of SV40 T-ag gene in the cells was confirmed by RT-PCR (Figure 2).

### Experiment 2. Effect of cytokines on production and content of Arachidonic Acid metabolites in immortalized bovine luteal endothelial cells (EnCL-1)

#### Experiment 2.1. Effect of TNFα and ifNγ on the viability of immortalized bovine luteal endothelial cells (EnCL-1)

**Figure 3:**
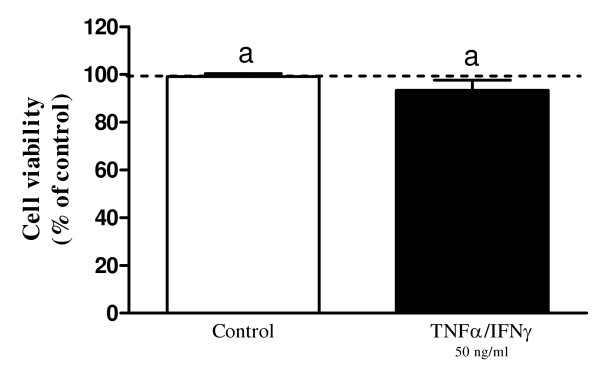
**Viability of non-treated and cytokine (TNFα/IFNγ) stimulated EnCL-1 cells**. No statistical differences were observed between groups of non-treated and cytokine treated cells (P > 0.05), as determined by one-way ANOVA followed by t-student's multiple comparison test.

TNFα/IFNγ did not influence the viability of EnCL-1 cells after 24 h of incubation comparing to non-treated cells (P > 0.05; Figure 3).

#### Experiment 2.2. Effect of TNFα and ifNγ on mRNA and protein expression of LTC_4 _synthase, LTA_4 _hydrolase, PGE_2 _synthase, PGF_2α _synthase and endothelin-1 in bovine endothelial immortalized cells (EnCL-1)

**Figure 4:**
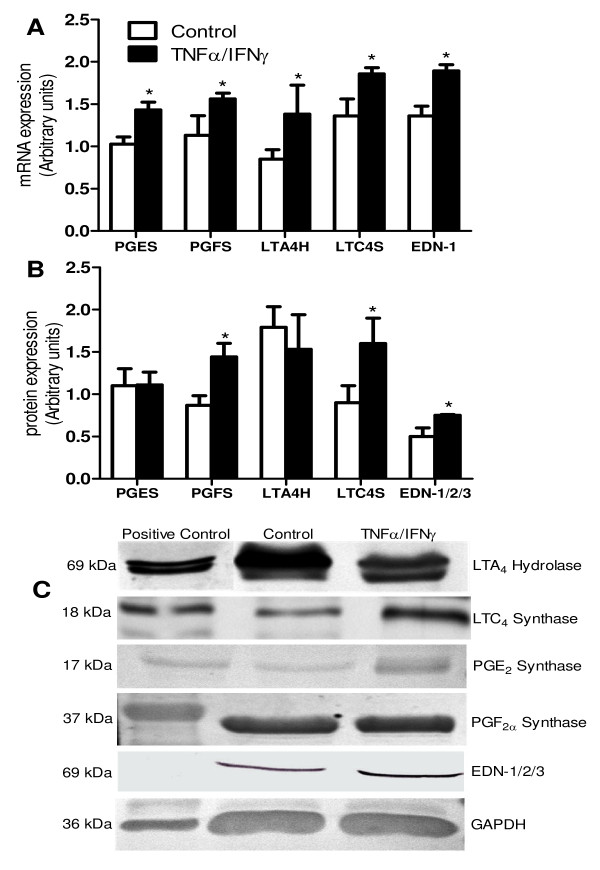
**mRNA and protein expression for selected aa metabolites in non- and cytokine treated EnCL-1 cells**. LTC_4 _synthase, LTA_4 _hydrolase, PGE_2 _synthase, PGF_2α _synthase and EDN-1 (A) mRNA, (B) protein expression of non-treated and cytokine (TNFα/IFNγ) stimulated EnCL-1 cells and (C) representive immunoblots of studies factors: 1 lane: Positive Control-bovine lung tissue, 2 lane-Control, 3 lane-TNFα/IFNγ. Arrows indicate statistical differences in the respective mRNA quantitative expression between groups of non-treated and cytokine treated cells (P < 0.05), as determined by one-way ANOVA followed by t-student's multiple comparison test.

TNFα/IFNγ treatment of EnCL-1 cells resulted in increased mRNA expression of PGES, PGFS, LTA4H, LTC4S and EDN-1 in comparison to untreated cells (P < 0.05; Figure 4A).

PGES and LTA4H protein expression were not affected by cytokine treatment (P > 0.05), whereas PGFS, LTC4S and EDN-1/2/3 protein expression were stimulated by cytokines (P < 0.05; Figure 4B). Representive immunoblots of studies factors are presented in Figure 4C.

#### Experiment 2.3. Effect of TNFα and ifNγ on prostaglandins, leukotrienes and endothelin-1 release by EnCL-1 cells

Cytokine treatment did not change the levels of PGE_2 _and LTB_4 _in the medium (P > 0.05), whereas cytokines stimulated PGF_2α_, LTC_4 _and EDN-1 release by EnCL-1 cells (P < 0.05; Table 2).

**Table 2:** Concentration of PGE_2_, PGF_2α__;_, LTB_4_, LTC_4 _and EDN-1 in non-treated and cytokine (TNFα/IFNγ) stimulated EnCL-1 cells (Mean ± SEM)

HORMONE	Control		TNFα/IFNγ	
(pg/2 × 10^5 ^cells)	Mean	SEM	Mean	SEM
PGE_2_	22,0	1,81	29,14	1,67
PGF_2α_	72,41	6,06	161,16***	8,69
LTB_4_	23,47	7,92	39,5	1,18
LTC_4_	116,86	10,96	709,5***	104,88
EDN-1	1005,5	187,58	4692,2***	946,34

Significant differences (*** - P < 0.001), as determined by one-way ANOVA followed by t-student's multiple comparison test (GraphPad PRISM).

## Discussion

The presence of SV40 T-ag in EnCL-1 cells and repeated passage without the apparent senescence confirmed the permanent status of the selected cell line. VE-cadherin and von Willebrand factor - positive features support the endothelial phenotype of the cell line. T antigen present in SV40 virus blocks cell death by apoptosis and it also interacts with a cytoplasmic protein that contains BH3 domain [33]. The viral genes achieve immortalization by inactivating the tumor suppressor genes (*p53, Rb*) that induce a replicative senescent state in cells. SV40 T antigen also induces telomerase activity in the infected cells [33]. So far, each study was conducted on the primary luteal endothelial cells or line of endothelial cells but not received from the bovine CL, which could possess different surface antigens and at least physiology [10]. We established the line of endothelial cells from bovine CL. To our knowledge, this is the first report showing the morphological and physiological properties of immortalized endothelial cells collected from bovine CL. According to Davis et al. [4], there are five types of bovine luteal endothelial cells differ the presence of cytokeratin, expression of surface antigens and neuronal cell adhesion molecule, capable for the contact between steroidogenic and endothelial cells within CL. We did not determine the morphological type and surface antigens of received line of endothelial cells than further study are necessary.

Endothelial cells posses receptors for TNFα [20,34,35] and IFNγ [36]. Several papers confirmed synergistic, antiproliferative and proapoptotic action of TNFα and IFNγ in the CL [4,10,19,21,22]. In this study, TNFα and IFNγ treatment of cells increased each of studied mRNA expressions. In addition, PGF_2α _secretion and its synthase protein expression were stimulated. Similar effect received Acosta et al. [8], TNFα elevated PGF_2α _content in fresh and unfrozen cells till 10^th ^passage in primary luteal endothelial cells. Whereas, in the study of Cavicchio et al. [10], cytokines stimulation was unresponsive to PGF_2α _secretion in the luteal endothelial cells (passage 2). The role of cytokines in regression of CL and cytokine effect on the main luteolytic factor-PGF_2α _was considered, as enhancing PGF_2α _action in the functional and structural luteolysis [17,19,20,34]. We also received the stimulation of mRNA expression for PGES without the effect on its protein expression and the level of PGE_2 _after cytokines treatment. Such an effect may be the consequence of changes in intracellular regulation of EnCL-1 cells, especially in mitochondrial activity. PGE_2 _enhances cellular proliferation, promotes angiogenesis, inhibits apoptosis and suppresses immune responses in cancerogenesis [35]. EnCL-1 cells potentially are programmed genetically for proliferation, thus cytokines, typically causing apoptosis, may not cause such an effect in our study and simultaneously stimulate PGE_2 _mRNA expression as kind of the preparation for proliferative functions.

Furthermore, we considered the role of cytokines in connection with another aa metabolites-LTs in EnCL-1 cells. mRNA expression, as well as protein expression for LTCS and LTC_4 _release were stimulated by cytokines in EnCL-1 cells. Cytokines also stimulated LTA4H mRNA expression but did not change LTA4H protein expression and LTB_4 _secretion in EnCL-1 cells. Recently we showed that primary bovine luteal endothelial cells show mRNA expression for LT receptors (for LTB_4 _and C_4_) [26]. So far LTs role in CL function focus on steroidogenic cells *in vitro *[30] or concerned with processes in bovine reproductive tract *in vivo *[37,38]. LTB_4 _plays luteotropic role in bovine CL, stimulating PGE_2 _secretion, whereas LTC_4 _stimulate PGF_2α _and thus acts as luteolytic factor *in vivo *[38]. There is lack of knowledge about LTs role in CL vascular processes like angiogenesis and angioregression. It is possible that cytokine effect in the ovary is modulated by LTs. The immune cells (macrophages, monocytes and leukocytes) infiltrate the ovary and secrete cytokines in process of ovulation [39]. Cytokines affect non-steroidogenic ovarian cells, causing the release of ovulation mediators, such as metabolites of aa: PGs and LTs [39]. Thus, cytokines are involved in ovarian processes during the estrous cycle such as differentiation of CL, ovulation, luteolysis and cooperate with PGs and LTs [26,30,37-39]. Beside, both PGs and LTs appear to act in parallel in the regulation of cell proliferation and neoangiogenesis [40].

We selected EDN-1 as one of the main factors produced in endothelial cells and checked the effect of cytokines action on edn-1 mRNA expression and EDN-1 release in EnCL-1 cells. Our results confirmed the earlier studies [8,20,41] because the cytokines stimulated both mRNA and its production in EnCL-1 cells. Protein expression of EDN-1/2/3 was also elevated in our study as summary of the expression for EDN-1, EDN-2 and EDN-3. EDN-1 mRNA and protein is expressed in luteal endothelial cells during all the estrous cycle and EDN-1 inhibits P4 production in late luteal phase [42,43], EDN-2, in the early CL [44], whereas EDN-3, on the contrary to mentioned EDN-1 and EDN-2, does not affect luteal steroidogenesis [45]. Our results indicate that cytokines enhance EDN-1/2/3 action and indicate on multiple characteristic functions of EnCL-1 cells.

Concluding, cytokines modulate EnCL-1 cells function by up-regulation of PGES, PGFS, LTA4H, LTC4S and mRNA expression. Protein expression was elevated by cytokines for PGFS and LTCS and simultaneously the level of appropriate active metabolites of these synthases products (PGF_2α _and LTC_4_) were higher after cytokine stimulation. Protein expression for PGES and LTA4H was not changed and release of products of these syntases-PGE_2 _and LTB_4 _was also stable. Beside, mRNA expression, level of EDN-1 and protein expression for EDN1/2/3 were upregulated by cytokines, which suggest that EnCL-1 cells show multiple potency, both prolifereative and proapoptotic. In this respect, EnCL-1 cells may serve as an appropriate model for investigating the paradigm of counteraction of the luteolytic and luteotropic properties of bovine CL. The received line of immortalized EnCL-1 cells possess stable genotype and phenotype. However, an intricate molecular structure of the cells with multiple intervenient factors/hormones and actions deserve to be defined.

## Competing interests

The authors declare that they have no competing interests.

## Authors' contributions

AJK collected the experimental material, isolated primary endothelial CL cells, carried out the immunoassays, mRNA expression and MTT methods and performed the statistical analysis, designed, drafted and coordinated the study. GB carried out the immortalization of the primary endothelial cells, participated in the design of the study and draft the manuscript. JB assisted and helped technically in all steps of experimental procedures. AB and DJS helped to design and draft the manuscript. All authors read and approved the final manuscript.
